# Sneddon Syndrome: A Case Report From Saudi Arabia

**DOI:** 10.7759/cureus.55509

**Published:** 2024-03-04

**Authors:** Abdulaziz Madani, Hanadi M Almutairi, Yazeed H Alshathry, Turki Albinhar, Maha M Barakeh

**Affiliations:** 1 Dermatology, King Saud University Medical City, Riyadh, SAU; 2 Department of Dermatology, King Saud University Medical City, Riyadh, SAU; 3 College of Medicine, King Saud University, Riyadh, SAU

**Keywords:** case report, vasculopathy, antiphospholipid antibodies, stroke, livedo racemosa, sneddon syndrome

## Abstract

Sneddon syndrome, also known as livedo reticularis with cerebrovascular accidents, is a rare but chronic condition that affects blood vessels in the skin and brain. This syndrome is characterized by a net-like appearance on the skin, known as livedo reticularis, which occurs due to the constriction of blood vessels. In addition to skin manifestations, Sneddon syndrome is often associated with repeated neurological events, such as strokes or transient ischemic attacks. These neurological symptoms can vary in severity and can lead to various complications. Upon admission to the stroke unit, a 28-year-old female was found to have bilateral livedo reticularis affecting the soles and the dorsal sides of the hands. Patient evaluation is done through medical history, physical examination, routine laboratory tests, and other diagnostic procedures.

## Introduction

Sneddon syndrome (SS) is a rare chronic condition that affects blood vessels, leading to a variety of symptoms. It mainly occurs in young adults between the ages of 20 and 42 years, with women being more commonly affected than men [1]. SS is characterized by a combination of the following two main features: livedo racemosa (LR) and neurological symptoms [2]. Reticularis is changed to racemosa. LR is a dermatological condition characterized by the presence of a persistent, erythematous, or violaceous discoloration on the skin, appearing in a branching, fragmented, and irregular pattern [3]. Neurological symptoms associated with SS can vary from person to person. Some individuals may experience migraines or frequent headaches, while others may exhibit even more severe manifestations, such as strokes, seizures, or cognitive impairment [1]. Although LR serves as a significant diagnostic indicator, neurological symptoms can occur before the onset of LR [4,5]. Although the exact cause of SS remains unclear, research has indicated that genetic factors, autoimmune responses, and coagulation disorders may contribute to its development [1]. This condition is frequently associated with retinal changes, cardiac valve involvement, and systolic labile hypertension. Risk factors suspected to be linked to SS include the use of estrogen-containing contraceptives, pregnancy, smoking, diabetes mellitus, atherosclerosis, and hyperlipidemia [5]. Diagnosis may involve a thorough evaluation of medical history, a physical examination, blood tests, imaging studies, and sometimes a biopsy of the affected skin.

## Case presentation

**Figure 1:**
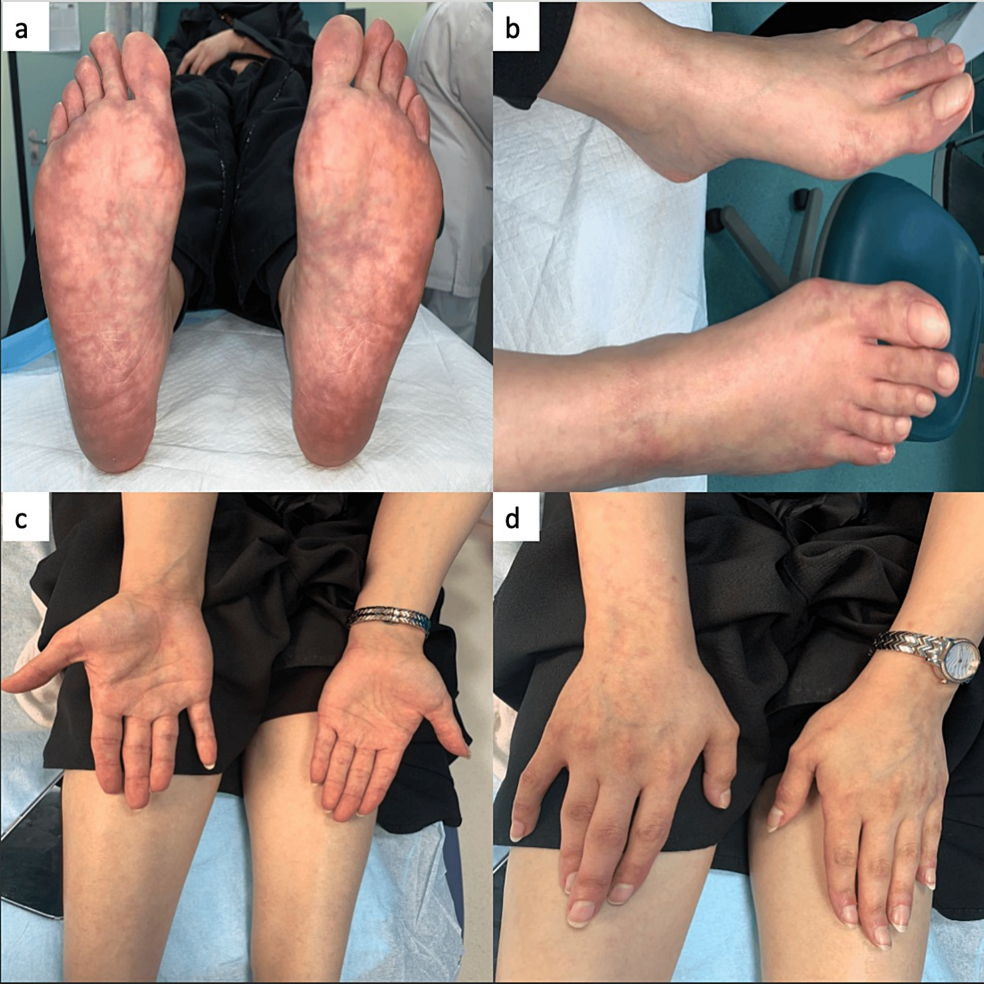
Multiple erythematous-to-violaceous irregular reticular patterns with incomplete circular patches (livedo racemosa) on both the plantar and dorsal aspects of her feet as well as the palmer and dorsal aspects of both hands.

A 28-year-old female presented with a sudden onset of symptoms, including weakness in her right arm and hand, facial asymmetry, and slurred speech. Upon examination, she had multiple erythematous-to-violaceous irregular reticular patterns with incomplete circular patches on both the plantar and dorsal aspects of her feet as well as the palmer and dorsal aspects of both hands (Figure 1). Pulmonary and cardiac auscultation were normal. Her blood pressure was: 117/70 mmHg. Neurological assessment revealed difficulties with naming and reading; nevertheless, comprehension, repetition, and writing abilities were intact. The pinprick sensation was slightly reduced in the right V2 and V3 compared to the left side.

**Figure 2:**
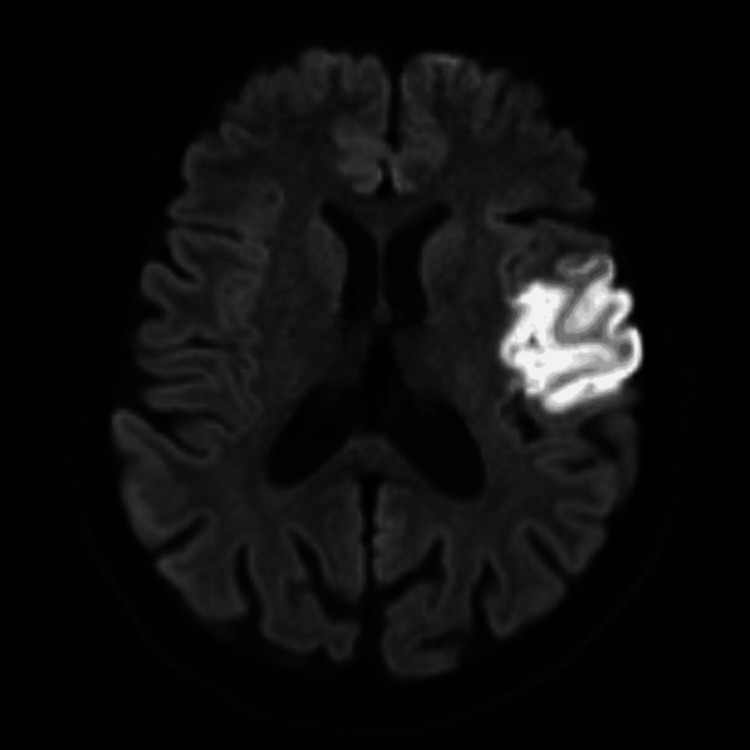
Brain magnetic resonance imaging showing an acute ischemic infarct in the left middle cerebral artery territory, affecting the insula and the frontoparietal lobe and extending to the pre- and post-central gyri.

The patient had a history of an ischemic stroke seven months earlier, specifically in the left middle cerebral artery, resulting in right-sided hemiplegia. She also experienced Raynaud’s phenomenon and exhibited a mild deficiency in protein S. Her current medications included aspirin (81 mg) and atorvastatin (20 mg) for the ischemic stroke, as well as sildenafil (25 mg) and amlodipine (5 mg) for Raynaud’s phenomenon. The differential diagnosis was protein S deficiency, antiphospholipid syndrome, vasculitis, SS, oral contraceptive pill use, and livedo reticularis. Her complete blood count, coagulation profile, and renal and liver functions were all within normal ranges. Laboratory results were negative, including autoimmune antibodies (antinuclear antibodies, antineutrophilic cytoplasmic antibody, Smith antibody, SS antibody, dsDNA ab, JO-1 antibody, cardiolipin antibody, β2-glycoprotein I antibody, myeloperoxidase, PR-3, and C3 and C4), hemophilia screening (*JAK2 *mutation, *FVL *mutation, *FII *mutation), and infectious disease screening (syphilis, Venereal Disease Research Laboratory cerebrospinal fluid (CSF), *Brucella *CSF, hepatitis B and C, and human immunodeficiency virus). Vasculitis screening including protein S level was found to be low, but protein C was normal. Direct immunofluorescence studies showed negative results for IgG, IgM, IgA, and C3. Fibrinogen showed scattered positivity without specific indications of vasculitis or thrombotic vasculopathy. A stroke workup was unremarkable for transthoracic echocardiography, transesophageal echocardiography, renal artery Doppler, and 48-hour Holter monitoring. A computed tomography (CT) scan showed no evidence of acute, well-established territorial infarction or intracranial hemorrhage, and CT angiography showed no filling defects. Brain magnetic resonance imaging (MRI) performed seven months earlier revealed an acute ischemic infarct in the territory of the left middle cerebral artery, affecting the insula and the frontoparietal lobe and extending to the pre- and post-central gyri. Moreover, characteristics of chronic small-vessel disease were noted (Figure 2).

**Figure 3:**
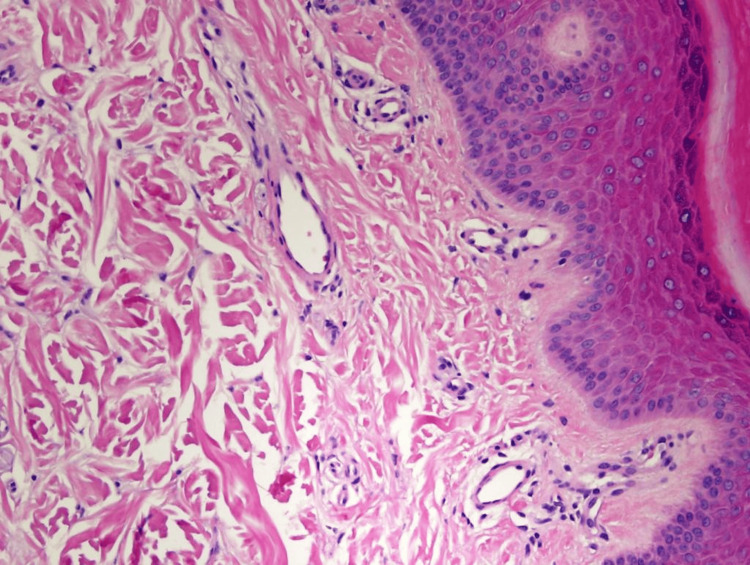
High-power photomicrograph of the epidermis and papillary dermis showing no significant pathological changes (hematoxylin and eosin stain, original magnification 400×).

A microscopic examination of a punch biopsy taken from the patient’s right foot confirmed the presence of normal and patent small dermal blood vessels (Figure 3).

**Figure 4:**
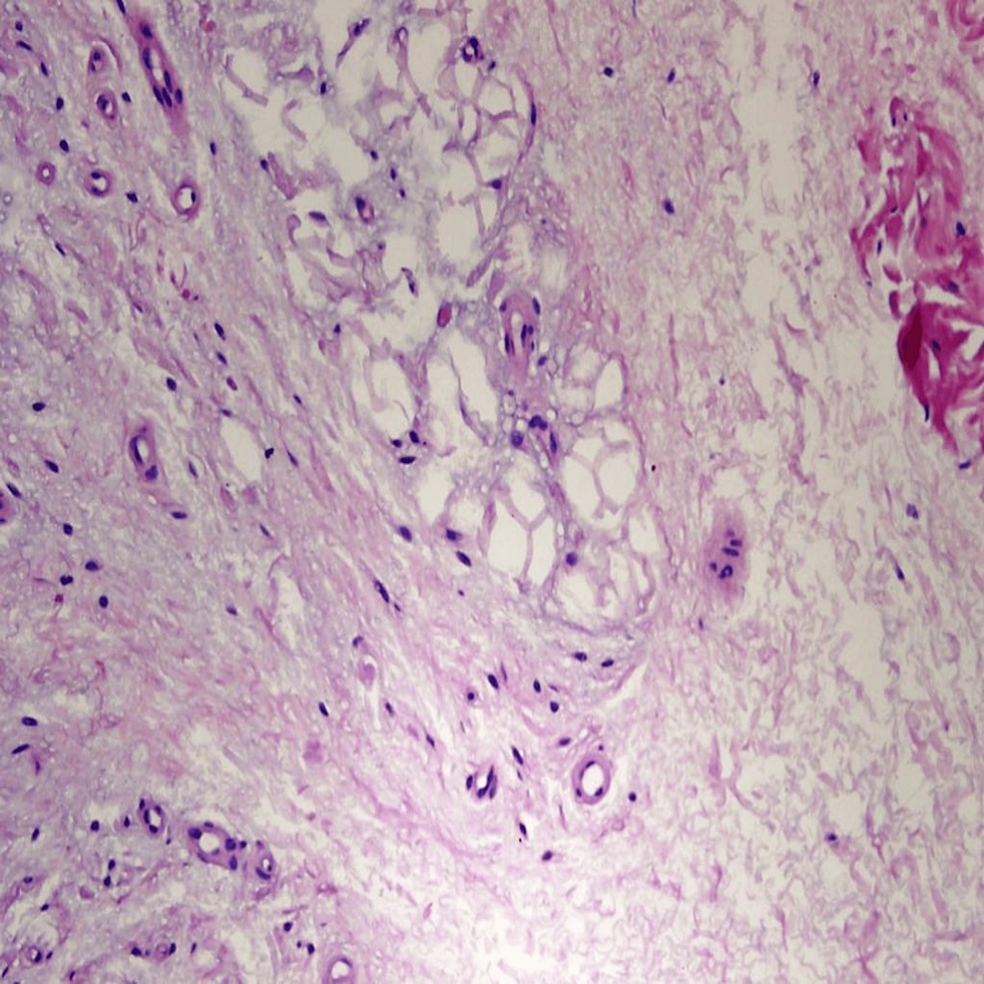
High-power photomicrograph of a skin punch biopsy showing focal fat necrosis (hematoxylin and eosin stain, original magnification 400×). No active inflammation or vasculitis was seen.

However, focal subcutaneous fat necrosis indicated ischemic changes (Figure 4).

A few months after this event, the patient had a miscarriage at 11-12 weeks of pregnancy as well as another attack of limb weakness and numbness. The patient is currently being followed by the neurology clinic, with no source of stroke having been identified at present.

Nifedipine, simvastatin, aspirin (81 mg), and angiotensin-converting enzyme inhibitors were used along with cyclophosphamide for eight months in a patient with seronegative SS and cognitive and behavioral decline. The patient demonstrated a subsequent improvement in subjective and objective memory and emotional status. Therefore, we propose that cyclophosphamide should be considered among first-line treatment options for patients with SS and cognitive impairment.

## Discussion

SS is a rare vasculopathic disorder. Its clinical manifestations include cerebrovascular disorders with LR. The incidence of this syndrome is approximately 4 per 1 million annually, and it favors young women [1]. SS can be classified into the following three categories: primary (no causative factor identified), autoimmune (with antiphospholipid antibodies or coexisting systemic lupus erythematosus or lupus-like disease), and thrombophilic [6]. The disorder mainly occurs sporadically; however, few familial cases have been reported [7]. SS has multiple systemic and dermatologic manifestations. On the skin, LR is defined as an erythematous-to-violaceous, irregular, discontinuous net-like pattern, and this condition may precede the onset of stroke by years [3]. The trunk or buttocks are involved in nearly all patients [1]; however, our case demonstrated a rare location of LR over the acral areas. Rarely, LR appears after neurological symptoms [5]. Another sign of SS on the skin is Raynaud’s phenomenon involving the hands and feet.

Stroke is a further diagnostic hallmark of SS which occurs due to ischemia [1]; hemiparesis, sensory disturbances, aphasia, and visual field defects are the most common clinical signs. Cognitive impairment and depression can occur in approximately 77% of SS patients [8]. Intracerebral, subarachnoid, or intraventricular hemorrhages are unusual in SS. Hypertension occurs in 15-65% of SS patients. Ischemic heart disease has also been described. In addition, 65% of SS patients show decreased levels of creatinine clearance [9]. Spontaneous abortion has also been associated with SS. A previous study found that 32 (84%) of 38 patients with SS were female. Of these patients, three had frequently miscarried, seven had suffered miscarriages for unclear reasons, and three women had experienced problems with sterility [10].

Management of SS focuses on controlling symptoms and preventing further complications. Although no cure is currently available, a multidisciplinary approach including medications to prevent blood clots, control blood pressure, and reduce inflammation is often adopted [5]. Controlling potential risk factors by maintaining a healthy weight, avoiding smoking, and engaging in regular exercise is advisable. Although coping with SS may be challenging, proper medical care and support can lead to satisfactory outcomes. Ongoing research continues to improve the understanding of this condition and explore new treatment options, offering hope for those affected by SS.

## Conclusions

SS is a rare neurocutaneous vasculopathy that has a debilitating effect on quality of life. LR, along with recurrent neurovascular events, are the hallmarks of the disorder. While the exact cause of SS remains unclear, ongoing research has suggested potential autoimmune or genetic factors. Effective management requires a multidisciplinary approach that involves medical intervention, lifestyle adjustments, and regular monitoring. Early diagnosis and treatment can lead to fulfilling lives and minimize the impact of this condition.
